# Core Microbiome of Slovak Holstein Friesian Breeding Bulls’ Semen

**DOI:** 10.3390/ani11113331

**Published:** 2021-11-22

**Authors:** Juraj Medo, Jana Žiarovská, Michal Ďuračka, Eva Tvrdá, Štefan Baňas, Michal Gábor, Matúš Kyseľ, Miroslava Kačániová

**Affiliations:** 1 Faculty of Biotechnology and Food Sciences, Institute of Biotechnology, Slovak University of Agriculture, Tr. A. Hlinku 2, 949 76 Nitra, Slovakia; juraj.medo@uniag.sk; 2 Faculty of Agrobiology and Food Resources, Institute of Plant and Environmental Sciences, Slovak University of Agriculture, Tr. A. Hlinku 2, 949 76 Nitra, Slovakia; 3 Faculty of Biotechnology and Food Sciences, Institute of Applied Biology, Slovak University of Agriculture, Tr. A. Hlinku 2, 949 76 Nitra, Slovakia; michal.duracka@uniag.sk (M.Ď.); eva.tvrda@uniag.sk (E.T.); xbanas@uniag.sk (Š.B.); 4 Faculty of Agrobiology and Food Resources, Institute of Nutrition and Genomics, Slovak University of Agriculture, Tr. A. Hlinku 2, 949 76 Nitra, Slovakia; michal.gabor@uniag.sk; 5 Research Centre AgroBioTech, Laboratory of Agrobiodiversity and Genetic Technologies, Slovak University of Agriculture, Tr. A. Hlinku 2, 949 76 Nitra, Slovakia; matus.kysel@uniag.sk; 6 Faculty of Horticulture and Landscape Engineering, Institute of Horticulture, Slovak University of Agriculture, Tr. A. Hlinku 2, 949 76 Nitra, Slovakia; miroslava.kacaniova@uniag.sk; 7 Department of Bioenergetics, Food Analysis and Microbiology, Institute of Food Technology and Nutrition, University of Rzeszow, Cwiklinskiej 1, 35-601 Rzeszow, Poland

**Keywords:** 16S rRNA sequencing, semen, Holstein Friesian breeding bulls

## Abstract

**Simple Summary:**

The aim of this study was to characterize the bacterial profile of semen collected from Holstein Friesian breeding bulls via a high-throughput sequencing approach for a 16S rRNA gene variability analysis. A total of 55 fresh semen samples of sexually mature breeding bulls were used in the study. They were gathered from Holstein Friesian breeding bulls at Slovak Biological Services in Nitra, Slovak Republic. To amplify the V4 region of the 16S rRNA bacterial gene, universal primers 515F and 806R enhanced by a 6 bp barcode identification sequence were used. The 16S rRNA high-throughput sequencing strategy was used. Two microbial clusters were identified among the analyzed samples—the first cluster was based on *Actinobacteria* and *Firmicutes,* while the second cluster contained a high prevalence of *Fusobacteria*.

**Abstract:**

Bacterial contamination of semen is an important factor connected to the health status of bulls that may significantly affect semen quality for artificial insemination. Moreover, some important bovine diseases may be transmitted through semen. Up to now, only a very limited number of complex studies describing the semen microbiome of bulls have been published, as many bacteria are hard to cultivate using traditional techniques. The 16S rRNA high-throughput sequencing strategy allows for the reliable identification of bacterial profiles of bovine semen together with the detection of noncultivable bacterial species. Fresh samples from Holstein Friesian breeding bulls (*n* = 55) were examined for the natural variability in the present bacteria. Semen doses were selected randomly from Slovak Biological Services in Nitra, Slovak Republic. The most predominant phyla within the whole dataset were *Firmicutes* (31%), *Proteobacteria* (22%), *Fusobacteria* (18%), *Actinobacteria* (13%) and *Bacteroidetes* (12%). Samples of semen were divided into two separate clusters according to their microbiome compositions using a cording partition around a medoids analysis. Microbiomes of the first cluster (CL1) of samples (*n* = 20) were based on *Actinobacteria* (CL1 average = 25%; CL = 28%) and *Firmicutes* (CL1 = 38%; CL2 = 27%), while the second cluster (CL2; *n* = 35) contained samples characterized by a high prevalence of *Fusobacteria* (CL1 = 4%; CL2 = 26%). Some important indicator microbial groups were differentially distributed between the clusters.

## 1. Introduction

Fertilization in the cattle industry is a complex of different multicomponent cascades and processes that are associated with various factors based on genetic, health and environment circumstances [1,2,3]. With its maximum of one pregnancy per year, reproduction of bovine species is less efficient in comparison to other livestock [4]. A broad spectrum of issues may affect the resulting fertility such as environmental causes, improper handling, transportation and storage of semen that deteriorates the final semen’s quality [5,6,7]. All of these factors are reported to be in great association with a decreased fertility; however, other aspects seem to be important for flawless assisted reproduction—such as bacterial contamination of semen [8].

The microbiological checkup of ejaculates as well as doses used for artificial insemination (AI) have become an inevitable part of strategies to eliminate the prevalence of bacteriospermia, which is reported to oscillate in a quite wide range from 7% up to 99% of ejaculates [9,10,11,12,13]. Variable biological groups of bacteria were identified in semen doses such as *Bacterioidetes*, *Actinobacteria*, *Proteobacteria*, *Firmicutes*, *Fusobacteria* or *Cyanobacteria* by classical methods [14]. Some opportunistic pathogens such as *Staphylococcus*, *Streptococcus*, *Mycoplasma*, *Pseudomonas*, *Corynebacterium* or *Bacillus* may even be present in ejaculates of clinically healthy bulls [15]. Naturally, such a wide range is closely associated with a physiological variability within species, breeds, age or season [16,17] as well as with the breeding management [9]. Ejaculates of some species were reported, on the other hand, to possess certain antibacterial defense mechanisms to mitigate potential bacterial contamination of the female genital tract in case a compromised semen sample is used for insemination [18,19]. An accurate and effective prediction of the fertility of bulls is of high importance, as it determines the economic parameters and the sustainability of the cattle industry [20] as the bacterial contamination of extended semen is reported to be a reason for sperm agglutination, acrosomal damage, decreased sperm motility or viability [21,22,23,24]. Currently, the bovine urogenital microbiome is has not been fully described or understood, especially in the case of males [25]. A previous study on the identification of bacterial presence in the semen of Holstein Friesian bulls used blood agars, Gassner agars and Tryptic soy agars, identifying the following bacteria: *Bacillus cereus, Staphylococcus cohnii, Staphylococcus klosii, Micrococcus luteus, Bacillus licheniformis, Staphylococcus xylosus, Staphylococcus aureus, Staphylococcus warneri, Staphylococcus lentus, Staphylococcus epidermidis, Bacillus mycoides* and *Staphylococcus haemolyticus* [26].

Nevertheless, no core microbiome has been defined for bull semen. A common core microbiome characterizes the most widespread microbial taxa within a host population [27]. However, the exact threshold of core taxa frequency is variable among authors. Bjork et al. [28] defined core taxa to be present in at least 70% of the time series; however, thresholds from 30% to 95 % were used. As such, the phenomenon of core microbiomes is used mainly because of a great diversity and complexity, as well as very dynamic changes that exist in microbiomes. A new approach in the characterization of bacterial communities was introduced by high throughput sequencing. The term “metagenome” is usually defined as the collection of genomes and genes of the microorganisms from an environment [29] and the most powerful advent of this strategy lies in the possibility of decoding both culturable and unculturable species from the samples of interest [30,31,32]. Amplicon-based high-throughput sequencing, which is the base of metagenomics, targets a specific genomic region that is ubiquitous and discriminatory throughout the population of microorganisms that are of interest in the study [33,34]. The most common genomic targets for bacteria are 16S rRNA genes [35].

The aim of this study was to characterize the bacterial profile of semen collected from Holstein Friesian breeding bulls via a high-throughput sequencing approach for a 16S rRNA gene variability analysis.

## 2. Materials and Methods

### 2.1. Biological Material Sampling and Preparation

A total of 55 fresh semen samples of sexually mature breeding bulls were used in the study. Each sample was obtained from an individual bull, and the bulls were not mating with a cow. The samples were gathered from Holstein Friesian breeding bulls at Slovak Biological Services in Nitra, Slovak Republic, during winter and early spring of 2019 and 2020, respectively. The animals were 4–6 years old and were fed a standard diet based on green and cereal fodder, berseem, straw and concentrated mixtures. Water was supplied constantly. The animals were kept loose in individual enclosures with access to outdoor exercise. The animals were regularly examined by a veterinarian to ensure proper health conditions. To maintain the external sterility, the artificial vagina was sterilized before sample acquisition and high standards of hygiene were followed during the whole process of sample collection. Prior to semen collection, the animals were allowed to urinate and their external genitalia were properly washed to avoid contamination of the ejaculate. Single-use gloves were changed between each collection. The obtained semen samples were transported into the laboratory immediately in a thermos to maintain a constant temperature of 10 °C in the vials that were disinfected with absolute ethanol (99.8%; Centralchem, Bratislava, Slovak Republic). For subsequent experiments, the vials were kept in a sterile Class II laminar flow hood. Only samples with at least a 70% motility and 1 × 10^9^ sperm/mL were processed further (*n* = 55).

### 2.2. DNA Extraction and Illumina Library Preparation

Genomic DNA was extracted from the semen samples via a DNeasy UltraClean microbial DNA kit (Qiagen, Germantown, MD, USA). The quantitative and qualitative parameters (A260 and A280) of the extracted DNA were analyzed via the Nanodrop NanoPhotometer (Implen, Westlake Village, CA, USA). To amplify the V4 region of the 16S rRNA bacterial gene, universal primers 515F and 806R enhanced by a 6 bp barcode identification sequence were used [36,37]. PCR was performed on 30 µL with the following composition: 20 ng of DNA, 0.3 µM/mL^−1^ of each primer and KAPA HiFi HotStart ReadyMix (1×) (Kapa Biosystems, Wilmington, NC, USA) in a SureCycler 8800 thermal cycler (Agilent, Santa Clara, CA, USA). The following thermal profile was used: 90 s of denaturation at 98 °C, 35 cycles (15 s of denaturation at 98 °C, 15 s of annealing at 62 °C and 15 s of elongation at 72 °C) with a final elongation step of 120 s at 72 °C. The PCR products were checked on 2% agarose gels in a TBE buffer containing ethidium bromide and were purified with a PCR purification kit (Jena Bioscience, Jena, Germany). Concentrations of the obtained PCR products were measured using a Qubit 2.0 Fluorometer (ThermoScientific, Walthem, MA, USA). The DNA of the samples were adjusted to an equal concentration and pooled together. The adapters were attached via a Truseq LT PCR-free kit (Illumina, Berlin, Germany). A MiSeq Reagent Kit v3 (600-cycle) (Illumina, Berlin, Germany) was used for sequencing.

### 2.3. Data Processing

The obtained basic raw sequencing data were processed via the Seed 2 software [38] to identify individual samples according to primer barcode tag sequences. Sequences with an overall quality lower than Q30 were removed from subsequent steps. Successful sequences were deposited in the gene bank under the bioproject number PRJNA767193. Autonomous sequence variants (ASVs) were obtained via the DADA2 [39] denoising algorithm through the QIIME 2 (version 2019.4.0) suite [40]. The most abundant sequences in each OTU were identified via the Ribosomal Database Project Classifier (version 2.13) against the 16S rRNA database (training set number 18) at a confidence threshold of 70% [41], and all of the mitochondria ASVs and non-identified ASVs were filtered out. Diversity core metrics OTU richness, Shannon’s diversity index, Pielou’s evenness and weighted Unifrac distance were calculated using QIMEE. The ASV table, identification data and diversity metrics were statistically analyzed in R [42]. The partition around medoids (PAM) clustering algorithm in R (library cluster) was used to identify possible groups inside the sample cohort. Clusters were made using a Bray–Curtis and Unifrac distance with settings of 2 to 10 clusters, and the optimal number of clusters was determined according to a silhouette analysis. The variation in richness and diversity among the identified clusters of samples was assessed using ANOVA followed by a Tukey HSD. The structure of communities was compared using ANOSIM statistics (library vegan). The differences in the microbial group distribution were evaluated using the Wilcoxon test.

## 3. Results and Discussion

The sequencing of the bacterial metagenomic amplicon libraries prepared for bovine semen samples resulted in 565, 035 reads after Q30 quality filtering, i.e., 10,273 reads per sample (maximum = 22,735; minimum = 6705). After subsequent processing including denoising, chimera discarding and mitochondria removal, high-quality reads were assigned to 1206 ASVs. According to a rarefaction curve analysis and Good’s coverage, all samples contained a sufficient number of sequences and curves became saturated before 2000 reads. According to a prior diversity analysis, all samples were rarified to 2710 sequences, which was a minimal count per sample.

**Figure 1:**
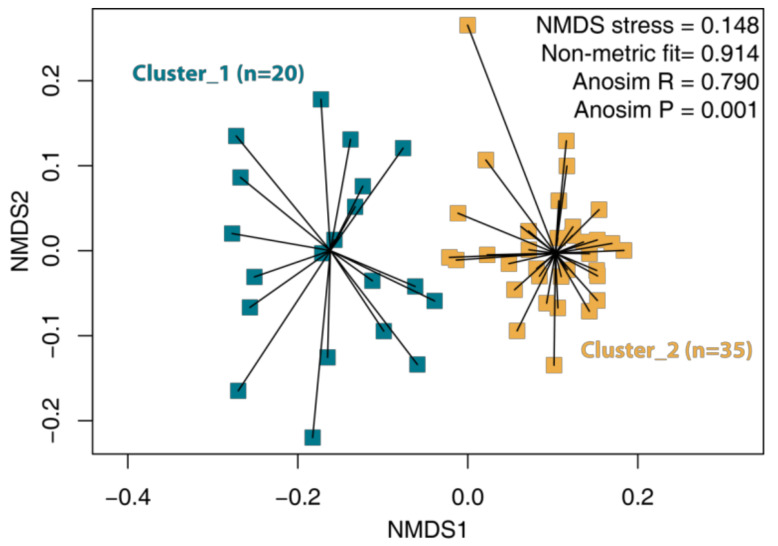
Nonmetric dimensional scaling (NMDS) scatterplot of semen samples acquired from clinically healthy breeding bulls. The samples were divided into two clusters according to the partition around a medoids (PAM) analysis.

A partition around medoids based on weighted unifrac distancies found two clusters to be the most suitable to describe the distribution of samples according to their ASVs (Figure 1). The structure of microbial communities between clusters of samples was significantly different according to the ANOSIM statistics (R = 0.790, *p* = 0.001). The differences between samples in these clusters are visible on all taxonomic levels; thus, we considered them as samples with truly separated types of semen microbiomes.

**Figure 2:**
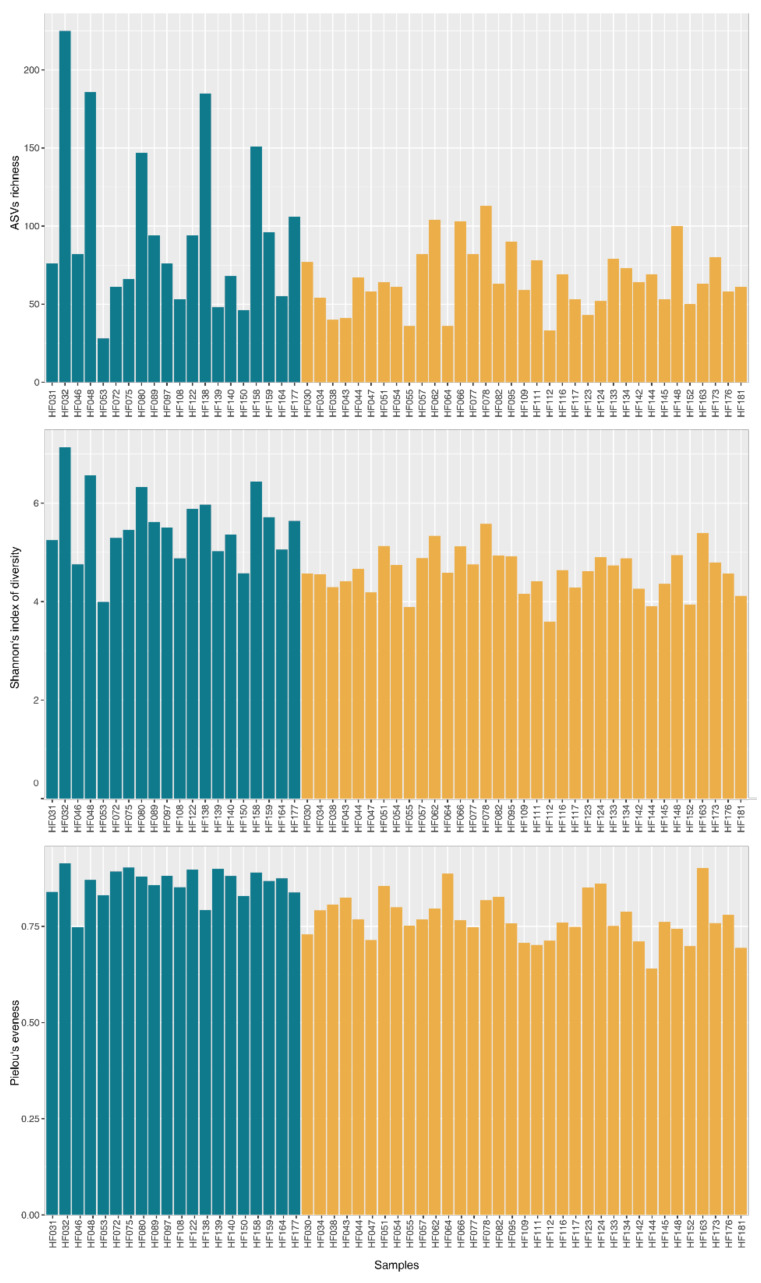
Diversity indices of microbial communities in the ejaculates of clinically healthy breeding bulls.

An analysis of the diversity indexes (Figure 2) confirmed differences between the sample clusters. Average richness (i.e., number of ASVs detected in a single sample) of the whole dataset was 77. While cluster 1 contained samples with 28–225 ASVs (mean = 97; SD = 54), cluster 2 contained samples with a richness in range of 33–113 (mean = 66; SD = 20) and their average values were significantly different (ANOVA *p* = 0.003). Similar differences were observed in the Shannon’s index where the mean value of cluster 1 was 5.52 (SD = 0.74) while the value of cluster 2 was 4.60 (SD = 0.45). The samples in cluster 2 also showed a significantly lower Pielou’s evenness than cluster 1 (0.77 (SD = 0.06) and 0.86 (SD = 0.04), respectively).

**Figure 3:**
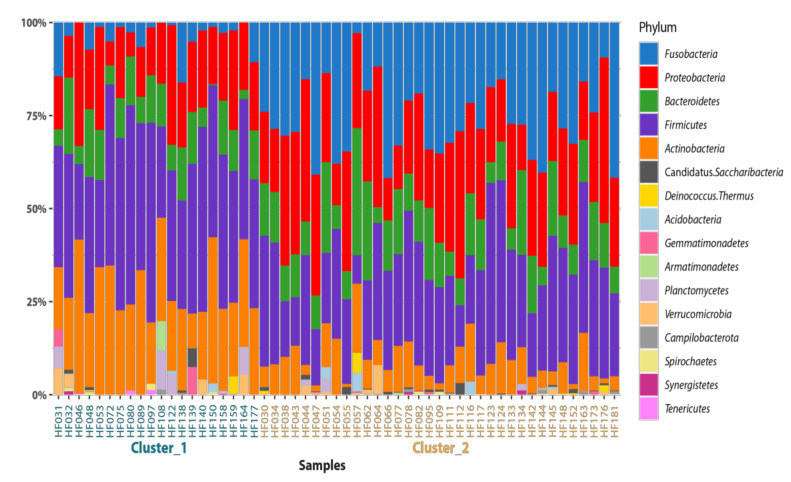
Relative abundance of bacterial phyla present in ejaculates of clinically healthy breeding bulls.

A total of 16 phyla were detected in the semen samples (Figure 3) while only five of them appear in at least one sample cluster in a frequency above 2%. According to Wilcoxon statistics, samples from cluster 1 contained significantly less Fusobacteria and Proteobacteria than the samples from cluster 2. Vice versa, the cluster 2 samples contained less Actinobacteria and Firmicutes representatives. A differential analysis performed on all taxonomic levels confirmed the statistically significant differences between clusters. All dominant groups (above 2% in any cluster) are listed in Table 1, and the summary statistic as well as the differences for the whole dataset and clusters are enclosed.

The same five dominant phyla were found as a part of microbiota in human seminal plasma: Proteobacteria, Firmicutes, Actinobacteria, Bacteroidetes and Fusobacteria [43]. This is also consistent with the findings of a previous study of the human testicular microbiome [44].

**Figure 4:**
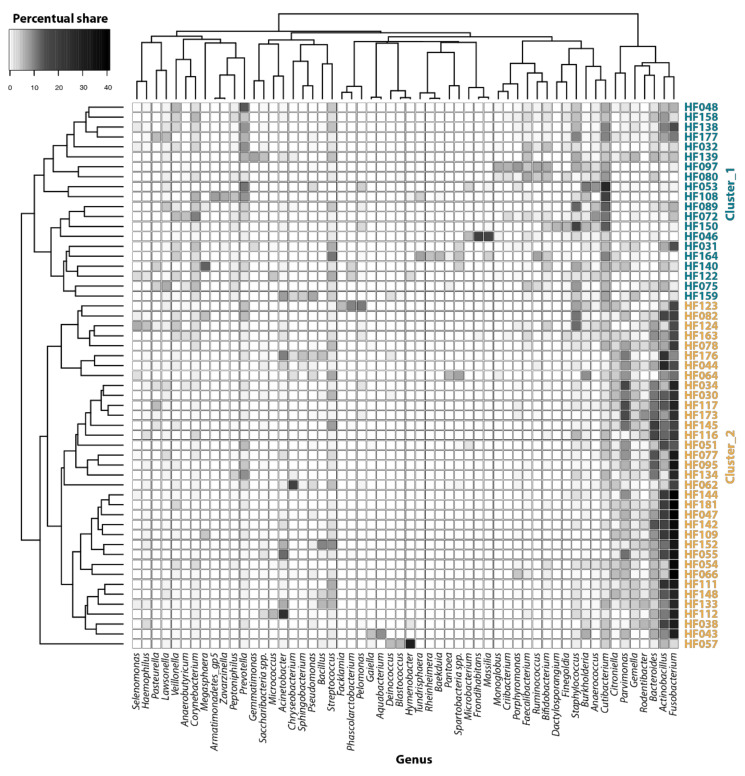
Heatmap of the most abundant bacterial genera (at least 5% of any sample) present in the ejaculates of clinically healthy breeding bulls.

Among the most prevalent genera, the fusobacterium *Actinobacillus* and Bacteroides were detected in cluster 2 samples while *Cutibacterium*, Staphylococcus or *Prevotella* were more abundant in cluster 1 (Figure 4). Other bacteria such as Streptococcus or Burkholderia were uniformly distributed among the samples.

Semen collection without a potential contamination with bacteria from the urogenital tract requires special aseptic procedures (i.e., testicular sperm aspiration) that are not usually used in animal breeding. As such, semen is often contaminated by microorganisms, and it is difficult to distinguish the source of certain members of the microbiome [45].

The microbiome composition obtained in this study is similar to the preputial microbiome of bulls recorded by [35]. The microbiome identified in our study was also divided into two groups according to the diversity indices, revealing groups diverse in their composition where clusters with a higher diversity frequently contained Fusobacteria. The authors also concluded that the penile microbiomes of bulls include members that are commonly found in soil, cow vaginas, respiratory tracts and feces, while it appeared that the microbiome was not affected by the diet, breed, age, farm or breeding history. In light of the mentioned facts and our results, the semen microbiome seems to be highly correlated with microbiomes present in other parts of the urogenital tract.

Besides commensal bacteria present in bovine ejaculates, pathogens could potentially be transmitted via bovine semen while being an inevitable part of the whole microbiome in different portions. The most refereed potential bacterial pathogens transmitted via semen belong to the genera Campylobacter, Brucella, Leptospira, Coxiella, Histophilus, Ureaplasma, Mycobacterium, Chlamydia and Mycoplasma [46].

Some members of the genus Campylobacter were detected in 5 of 20 samples from cluster 1 and in 9 of 35 samples in the cluster 2. However, their share of the total microbiome remains low, with a maximum frequency of 1% in sample HF144. Campylobacteriosis is reported as a venereal disease with a worldwide distribution [47]. Reporting this pathogen in this metagenomic study of the samples collected from healthy bulls reanalyzes previous knowledge about the role of bulls in spreading venereal diseases while remaining asymptomatic [48,49,50]. The 16S rDNA metagenomic approach was applied in a case of human sperm microbiota where three main interaction bacterial modules were found, with Campylobacter being a part of one module together with other strictly anaerobic genera such as Prevotella, Finegoldia, Actinomyces, Fusobacterium, Dialister and Peptoniphilus [51], and it is reported to be a bacterial species associated with clinical criteria in a significant manner [52].

Haemophilus was detected in seven samples from cluster 1 and in 21 samples from cluster 2. A total of four ASVs were identified as Haemophilus, and ASV45 (99.5% similarity to Haemophilus paracuniculus according to BLAST) was found in 16 samples that belonged to cluster 2. Haemophilus was reported to be one of the most abundant bacterial genera in human semen samples along with Proteobacteria members [51]. With respect to the pathogen search, the Brucelaceae family was present in some samples. Although Brucella was present only in one sample (HF062; 0.5%), the other member of Brucelacea, from the genus Pseudochrobactrum, was detected in 13 samples from cluster 2. Pseudochrobactrum saccharolyticum was found in the seminal fluid collected from patients with prostate cancer or benign prostatic hyperplasia [53]. In some samples, the presence of Enterobacteriaceae was found. This taxon is one of the main contaminants of semen, and their presence may result in a lower sperm concentration and in lower motility as well as a higher percentage of spermatozoa with a damaged plasma membrane or acrosome [54]. Achromobacter was reported to be able to negatively affect a successful insemination process [55]. On the other hand, an important portion of identified taxa, Lactobacillales, was identified in the analyzed samples. These were more prevalent in cluster 1 (5%) than in cluster 2 (3.5%). Lactobacillales are considered indicators of a healthy microbiome of the urogenital tract, and their occurrence is typical for normospermic individuals [51].

The semen microbiome is reported to have an important impact on the qualitative parameters of sperm [51]. Different microorganisms are furthermore reported to play a role in sperm dysfunction [52]; however, studies on individual taxa are rather informative. Hence, complex correlations depicting the microbiome as a whole may provide more specific knowledge of biological relevance. Only a few information from metagenomic bacterial characterizations are reported, if any. Most metagenomic studies on the bovine reproductive tract are relevant for vaginal or uterine specimens [25]. The commonly identified bacterial phyla in bovine female reproductive tracts are represented by Bacteroidetes, Firmicutes and Proteobacteria, followed by Actinobacteria and Fusobacteria. Some of them correspond to the phyla that were detected in our study on bovine semen. Bacteria from these phyla were reported to be present regardless of the breed, farm, gender, geographical location, sampling site, reproductive status or reproductive health [25].

In the case of specific specimens, such as semen samples, it is still difficult to identify all bacteria using conventional microbiological techniques, particularly because, if the samples are cultured first, the colonies that are detected fully depend on the culture conditions. Furthermore, some bacteria are either slow or not growing at all, or may be in a competitive regime with other species [56,57]. Moreover, numerous bacteria cannot be differentiated via phenotypic identification. Another possibility, MALDI-TOF for bacterial identification, has increased considerably [58,59], but the identification ability in this case depends mainly on the information available in the database. The identification of isolates based on DNA sequences allows for precise identification; however, it requires advanced laboratory and bioinformatic skills, and thus, this technique is used mainly in the research field. Furthermore, recently, whole genome sequences have been used for the characterization of isolates.

The identification of noncultivable members of microbiomes relies on shotgun metagenomics or targeted loci (amplicon sequencing). The shotgun metagemic approach generates data that also describe the distribution of microbial functional genes, but it is significantly more expensive and requires an extensive bioinformatic analysis in comparison to amplicon sequencing. As 16S rRNA gene sequences constitute a base of the phylogenetic taxonomic system, practically all prokaryotic microorganisms, including the noncultivable ones, may be classified by an analysis of this gene. In the case of some species complexes, identification and classification should involve additional genes (multilocus analysis) to achieve a species level of resolution [60]. On the other hand, partial sequences usually used in high-throughput sequencing techniques are not sufficient to obtain a species-level classification for most microorganisms. In the future, the technique has the potential to be incorporated into veterinary laboratories as well [61], and despite its limitations, 16S sequencing is reported and considered to be the gold standard for bacterial identification [62].

## 4. Conclusions

Here, a typical bacterial community was described in the semen of healthy Holstein Friesian breeding bulls. Only limited information exists currently for this type of specimen. The analysis of 55 samples revealed the microbiome members typical of the urogenital tracts of bulls. Two different clusters were found among the samples. The first cluster is characterized by the presence of Firmicutes and Actinobacteria. The second cluster exhibits a high portion of Fusobacteria. More knowledge in this field may reinforce our understanding of the microbial communities typical of the bovine reproductive system and may help to manage assisted reproductive technologies in a more precise manner. 16S high-throughput sequencing seems to be a viable alternative to classical microbiological methods in the study of the bacterial composition of seminal fluid, not only in bulls but also in other species of domestic animals.

## Figures and Tables

**Table 1:** Core microbiome dominant groups (above 2% in any cluster) in Holstein Friesian semen samples. The table depicts the average microbiome share for each taxa in identified clusters accompanied with standard errors and differences in their distribution in clusters according to Wilcoxon statistics.

Phylum	Class	Order	Family	Genus	ASV	Average Share ± SE in Cluster 1	Number of Samples Detected in Cluster 1 (*n* = 20)	Average Share ± SE in Cluster 2	Number of Samples Detected in Cluster 2 (*n* = 35)	*p* Value
*Actinobacteria*						24.57 ± 1.87	20	7.74 ± 0.8	35	0.001
	*Actinobacteria*					23.55 ± 1.94	20	7.33 ± 0.74	35	0.001
		*Bifidobacteriales*				2.18 ± 0.45	17	0.53 ± 0.14	23	0.001
			*Bifidobacteriaceae*			2.18 ± 0.45	17	0.53 ± 0.14	23	0.001
				*Bifidobacterium*		2.17 ± 0.45	17	0.51 ± 0.14	23	0.001
		*Micrococcales*				3.76 ± 1.67	17	0.87 ± 0.19	32	0.003
			*Microbacteriaceae*			2.16 ± 1.59	13	0.19 ± 0.07	14	0.022
		*Mycobacteriales*				5.39 ± 0.85	20	1.78 ± 0.23	34	0.001
			*Corynebacteriaceae*			3.37 ± 0.56	18	1.13 ± 0.16	33	0.001
				*Corynebacterium*		3.37 ± 0.56	18	1.13 ± 0.16	33	0.001
		*Propionibacteriales*				9.88 ± 1.3	20	3.11 ± 0.35	35	0.001
			*Propionibacteriaceae*			9.31 ± 1.3	20	2.99 ± 0.35	35	0.001
				*Cutibacterium*		9.15 ± 1.28	20	2.85 ± 0.33	35	0.001
					*ASV7*	7.92 ± 1.03	20	2.69 ± 0.32	35	0.001
*Bacteroidetes*						10.17 ± 1.2	20	12.73 ± 1.14	35	0.267
	*Bacteroidia*					8.73 ± 1.26	20	10.12 ± 0.86	34	0.319
		*Bacteroidales*				8.73 ± 1.26	20	10.12 ± 0.86	34	0.319
			*Bacteroidaceae*			2.26 ± 0.53	16	7.65 ± 0.78	34	0.001
				*Bacteroides*		1.68 ± 0.42	14	7.42 ± 0.79	34	0.001
					*ASV4*	0.82 ± 0.33	9	6.47 ± 0.68	34	0.001
			*Prevotellaceae*			4.83 ± 0.99	18	1.81 ± 0.36	28	0.015
				*Prevotella*		4.31 ± 0.94	18	1.64 ± 0.34	28	0.028
*Firmicutes*						38.08 ± 2.1	20	26.63 ± 1.52	35	0.001
	*Bacilli*					12.18 ± 1.12	20	9.02 ± 0.87	35	0.027
		*Bacillales*				7.06 ± 1.06	20	5.47 ± 0.58	35	0.228
			*Bacillales_Incertae Sedis XI*			1.28 ± 0.38	14	2.23 ± 0.24	34	0.005
				*Gemella*		1.28 ± 0.38	14	2.23 ± 0.24	34	0.005
			*Staphylococcaceae*			5.19 ± 1.01	19	2.28 ± 0.48	34	0.005
				*Staphylococcus*		5.18 ± 1.01	19	2.27 ± 0.48	34	0.005
					*ASV9*	4.51 ± 0.95	19	1.96 ± 0.44	33	0.003
		*Lactobacillales*				5.12 ± 0.58	20	3.54 ± 0.49	35	0.025
			*Streptococcaceae*			4.07 ± 0.56	20	2.86 ± 0.41	34	0.033
				*Streptococcus*		3.33 ± 0.62	19	2.72 ± 0.41	34	0.354
	*Clostridia*					22.25 ± 2.35	20	15.24 ± 1.1	35	0.039
		*Clostridiales*				22.25 ± 2.35	20	15.24 ± 1.1	35	0.039
			*Lachnospiraceae*			6.08 ± 0.96	20	1.52 ± 0.3	32	0.001
			*Peptoniphilaceae*			7.45 ± 0.7	20	12.13 ± 0.92	35	0.002
				*Anaerococcus*		2.27 ± 0.57	17	0.51 ± 0.12	28	0.001
				*Citroniella*		0.64 ± 0.25	11	2.63 ± 0.32	33	0.001
					*ASV11*	0.46 ± 0.22	9	2.58 ± 0.31	33	0.001
				*Helcococcus*		0.39 ± 0.15	12	2 ± 0.21	33	0.001
				*Parvimonas*		0.99 ± 0.33	16	6.15 ± 0.76	35	0.001
					*ASV5*	0.99 ± 0.33	16	6.15 ± 0.76	35	0.001
			*Ruminococcaceae*			6.65 ± 1.45	19	0.95 ± 0.18	32	0.001
				*Faecalibacterium*		2.25 ± 0.48	18	0.4 ± 0.12	22	0.001
	*Negativicutes*					3.52 ± 0.93	15	2.19 ± 0.52	33	0.468
		*Veillonellales*				2.75 ± 0.69	14	1.34 ± 0.31	31	0.204
			*Veillonellaceae*			2.75 ± 0.69	14	1.34 ± 0.31	31	0.204
*Fusobacteria*						4.23 ± 1.05	18	26.28 ± 1.69	35	0.001
	*Fusobacteriia*					4.23 ± 1.05	18	26.28 ± 1.69	35	0.001
		*Fusobacteriales*				4.23 ± 1.05	18	26.28 ± 1.69	35	0.001
			*Fusobacteriaceae*			3.96 ± 1.05	18	26.13 ± 1.68	35	0.001
				*Fusobacterium*		3.96 ± 1.05	18	26.13 ± 1.68	35	0.001
					*ASV1*	1.73 ± 0.78	10	9.68 ± 1.23	34	0.001
					*ASV2*	0.13 ± 0.08	4	3.16 ± 0.53	29	0.001
					*ASV8*	0.91 ± 0.38	10	9.41 ± 1.28	34	0.001
					*ASV10*	0.75 ± 0.33	7	3.69 ± 0.47	32	0.001
*Proteobacteria*						18.14 ± 1.62	20	24.67 ± 1.42	35	0.007
	*Alphaproteobacteria*					2.3 ± 0.66	19	1.3 ± 0.46	30	0.041
	*Betaproteobacteria*					6.69 ± 1.36	20	3.54 ± 0.73	34	0.005
		*Burkholderiales*				5.43 ± 1.37	20	2.79 ± 0.71	34	0.016
			*Burkholderiaceae*			2.64 ± 0.77	20	1.54 ± 0.33	34	0.064
				*Burkholderia*		2.22 ± 0.6	20	1.47 ± 0.32	34	0.298
					*ASV17*	2.22 ± 0.6	20	1.45 ± 0.32	34	0.278
	*Gammaproteobacteria*					8.87 ± 1.46	20	19.72 ± 1.57	35	0.001
		*Pasteurellales*				5.23 ± 1.12	19	15.45 ± 1.29	35	0.001
			*Pasteurellaceae*			5.23 ± 1.12	19	15.45 ± 1.29	35	0.001
				*Actinobacillus*		2.99 ± 0.76	17	12.05 ± 1.17	35	0.001
					*ASV3*	1.7 ± 0.43	14	6.31 ± 0.69	34	0.001
					*ASV6*	1.29 ± 0.39	13	5.73 ± 0.6	34	0.001
				*Rodentibacter*		0.25 ± 0.13	6	2.11 ± 0.35	30	0.001
		*Pseudomonadales*				1.87 ± 0.76	16	3.25 ± 0.88	33	0.214
			*Moraxellaceae*			1.25 ± 0.48	14	2.6 ± 0.83	31	0.251
				*Acinetobacter*		0.76 ± 0.39	10	2.36 ± 0.82	25	0.089
					*ASV13*	0.63 ± 0.4	7	2.21 ± 0.83	20	0.086

## Data Availability

Sequences were deposited in the gene bank under the bioproject number PRJNA767193 with SRA SRR16352497-SRR16352443 in NCBI database.
